# Extremophiles in Mineral Sulphide Heaps: Some Bacterial Responses to Variable Temperature, Acidity and Solution Composition

**DOI:** 10.3390/microorganisms3030364

**Published:** 2015-07-09

**Authors:** Helen R. Watling, Denis W. Shiers, David M. Collinson

**Affiliations:** CSIRO Mineral Resources Flagship, Australian Minerals Research Centre, P.O. Box 7229, Karawara, WA 6152, Australia; E-Mails: Denis.Shiers@csiro.au (D.W.S.); David.Collinson@csiro.au (D.M.C.)

**Keywords:** heap bioleaching, bacterial acid stress, bacterial heat stress, bacterial metal tolerance

## Abstract

In heap bioleaching, acidophilic extremophiles contribute to enhanced metal extraction from mineral sulphides through the oxidation of Fe(II) and/or reduced inorganic sulphur compounds (RISC), such as elemental sulphur or mineral sulphides, or the degradation of organic compounds derived from the ore, biota or reagents used during mineral processing. The impacts of variable solution acidity and composition, as well as temperature on the three microbiological functions have been examined for up to four bacterial species found in mineral sulphide heaps. The results indicate that bacteria adapt to sufficiently high metal concentrations (Cu, Ni, Co, Zn, As) to allow them to function in mineral sulphide heaps and, by engaging alternative metabolic pathways, to extend the solution pH range over which growth is sustained. Fluctuating temperatures during start up in sulphide heaps pose the greatest threat to efficient bacterial colonisation. The large masses of ores in bioleaching heaps mean that high temperatures arising from sulphide oxidation are hard to control initially, when the sulphide content of the ore is greatest. During that period, mesophilic and moderately thermophilic bacteria are markedly reduced in both numbers and activity.

## 1. Introduction

**Figure 1:**
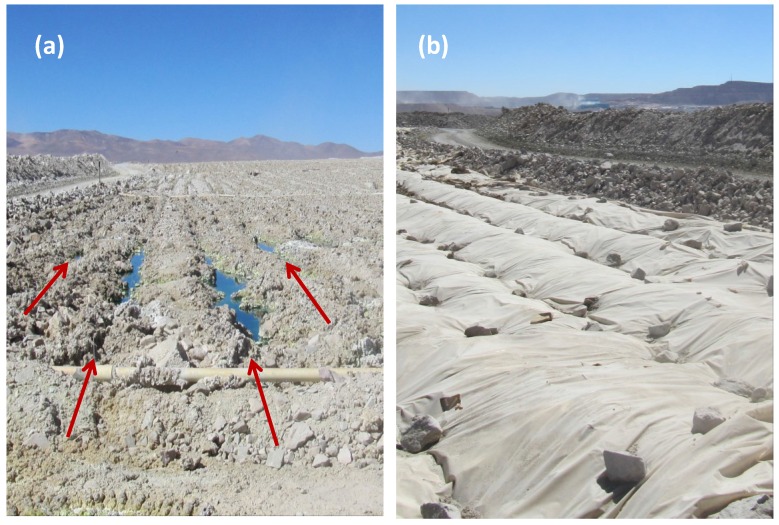
Run-of-mine dump bioleach at Escondida, Chile showing (**a**) surface with irrigation lines and (**b**) covers to retain heat and moisture. Arrows indicate the distribution and direction of irrigation lines, also evidenced by surface ponding.

Increasingly, mining companies are turning to lower grade and more complex ores to meet global demand for base metals, in particular copper. The preferred technology for metal extraction from low-grade ores is heap leaching for oxidised ores and heap bioleaching for sulphidic ores [1]. The technology is relatively simple and low cost and has been applied to copper, nickel, gold and uranium ores. Products include high-quality metals obtained using solvent extraction and electrowinning, or hydroxide or sulphide precipitates requiring further processing. In the case of copper, the Escondida (Chile) run-of-mine heap (ROM) leach is the largest “bioreactor” in the world [2] (Figure 1). The surface area of the overall leach pad is approximately 10^7^ m^2^, and ore is being stacked in 18-m lifts to a planned final height of 125 m. At the other end of the scale, the technology has also been implemented for small deposits in extreme climates, where heap pads are smaller, for example 20–60 × 10^3^ m^2^, and lift heights lower (3–10 m). Examples are copper heap leaching on the edge of the Great Sandy Desert, northwestern Australia [3], nickel/copper heap leaching in sub-arctic conditions [4] or copper leaching in the tropics [5]. Typical irrigation rates per square meter of surface are in the range 5–10 L·h^−1^.

In heap bioleaching, some acidophilic chemolithotrophic extremophiles contribute to the oxidation of mineral sulphides through the oxidation of Fe(II) (Equation (1)) and/or reduced inorganic sulphur compounds (RISC), such as elemental sulphur or mineral sulphides (Equations (2)and (3)) under aerobic conditions. Those with the ability to reduce Fe(III) under anaerobic or micro-aerobic conditions may also contribute by dissolving insoluble Fe(III) compounds to release target metals. Heterotrophs and mixotrophs contribute through the utilisation of organic compounds that may inhibit the activities of the chemolithotrophs (e.g., glucose, Equation (4)). Acidophilic or acid-tolerant extremophiles that participate in the iron and sulphur cycle have been listed [6], and those reported to colonise heaps have been summarised [7].

4 Fe^2+^ + O_2_ + 4 H^+^ → 4 Fe^3+^ + 2 H_2_O (1)

2 S° + 2 H_2_O + 3 O_2_ → 2 H_2_SO_4_ (2)

CuFeS_2_ + 4 Fe^3+^ → 5 Fe^2+^ + Cu^2+^ + 2 S° (3)

C_6_H_12_O_6_ + 24 Fe^3+^ + 6 H_2_O → 24 Fe^2+^ + 6 CO_2_ + 24 H^+^ (4)

Ideally, knowledge of microbial activities in mineral sulphide heaps should be gained using *in situ* probes and collecting samples from different spatial locations representative of the whole heap. However, the size of the “bioreactor” and the aggressive acidic environment tend to deliver incomplete results due to an insufficiency of monitoring sites and/or to probe malfunction due to corrosion in the acid environment. Even relatively simple solution sampling points (lysimeters) placed within the ore bed tend to become clogged with mobilised fine particles and precipitates. In addition, there is a reluctance to allow frequent sampling campaigns at commercial operations, as they are perceived to interrupt or hinder metal production. Therefore, the use of laboratory-generated data offers an indirect means of generating information that can be used in conjunction with metallurgical data to “describe heap biological health” and inform management operational decisions.

Customised monitoring methods for the three main microbial functions, ferrous-ion oxidation, RISC oxidation and carbon utilisation have been developed and comparative laboratory-scale data acquired using single strains of four common bacterial species detected in copper heap leach operations. The species are: *Acidithiobacillus* (*At.*) *ferrooxidans*, an obligate chemolithoautotrophic Fe(II)- and RISC-oxidiser that does not obtain carbon from organic compounds [8,9,10]; *Sulfobacillus* (*S.*) *thermosulfidooxidans*, a mixotrophic Fe(II)- and RISC-oxidiser [11]; *At.* *caldus*, an autotrophic RISC oxidiser that can grow mixotrophically using yeast extract or glucose [12,13]; or “*Alicyclobacillus* *(Ab.*) *cupritolerans*”, a heterotroph capable of Fe(II) oxidation [14]. Bacterial activities under controlled, but varied conditions were investigated to provide an overview of “biological health” related to heap conditions. The variables selected represent some key characteristics of heap environments, such as fluctuating temperatures due to sulphide oxidation, the consumption of acid leading to localised areas of low solution acidity or the impacts of solution components derived from the dissolution of copper and/or gangue minerals in recycled process water. Ancillary data pertinent to heap leaching have been taken from some of our bioleaching studies to provide context and to illustrate each of the topics. The overall study is ongoing, with the longer-term goal of developing a database of activities linked to heap conditions that can be used to inform heap management practice at commercial operations.

## 2. Materials, Methods and Data Treatments

All reagents used in this study were analytical grade (AR) unless otherwise stated, and solutions were prepared with deionised water. Preparation and transfer of sterile solutions, sub-culturing, inoculation of test flasks and sampling took place in a laminar flow hood using an aseptic technique.

### 2.1. Cultures and Culture Media

The bacterial strains used in this study were *At. ferrooxidans* DSM 14882^T^, *S. thermosulfidooxidans* DSM 9293^T^, *At. caldus* DSM 8584^T^ and *Alicyclobacillus* (*Ab.*)-like strain FP1, informally named “*Ab. cupritolerans*”. The strains had been maintained in the laboratory for up to five years in the appropriate salt media without contaminants or additives other than the selected substrates and yeast extract (as a growth factor). Strain purity was confirmed during the test work by sequencing the 16S rRNA gene.

Basal salt medium (BSM) contained (g·L^−1^): 1.5 (NH_4_)_2_SO_4_, 0.25 KH_2_PO_4_ and 0.25 MgSO_4_ in water adjusted to pH 1.8 with 18 M H_2_SO_4_. The medium was autoclaved (121 °C, 30 min). Except for tests with *At. ferrooxidans*, filter-sterilised yeast extract (YE) solution was added to the bulk medium (final concentration 0.1 g·L^−1^ YE). Sterile iron medium for Fe(II)-oxidation tests was prepared by aseptically removing a 20-mL aliquot from 1 L of BSM (or BSM-YE), dissolving 10 g FeSO_4_·7H_2_O in it and filter sterilising the solution back into the original medium bottle. Sterile solutions of tetrathionate medium for RISC oxidation tests (0.75 g·L^−1^ K_2_S_4_O_6_) were prepared in a similar manner. Other test elements were added to the media as sulphate salts. For growth on elemental sulphur, media were amended with 5.0 g·L^−1^ sterile sulphur. The sulphur was sealed in a bottle in which the head-space was filled with nitrogen gas, and then (three times) heated in an oven at 100 °C for 1 h and cooled.

### 2.2. Organic Carbon Utilisation

Chemical oxygen demand (COD) analyses were conducted on periodic samples from reactors to quantify the reduced carbon species present. Liquor samples were passed through a 0.45-μm pore-size membrane and 5.0 mL transferred to a 500-mL Schott bottle. Blank solutions (controls) were prepared with distilled water. A 10-mL aliquot of K_2_Cr_2_O_7_ solution (10 g·L^−1^) and 2 mL 18 M H_2_SO_4_ were added to the sample and the solution heated at 110 °C for 5 h in a bath containing polyethylene glycol. After cooling to room temperature, a back titration using 30 g·L^−1^ FeSO_4_·7H_2_O solution was conducted to an electrochemical end point.

### 2.3. Ferrous Ion Oxidation

A calibrated pH meter fitted with a glass membrane electrode was used to measure solution pH. The method used to monitor ferrous ion oxidation is based on that described by Pesic *et al.* [15]. It has been shown that the rate of product formation, such as sulphate from sulphur biooxidation or ferric ions from ferrous ion biooxidation, is proportional to the autotrophic growth of microorganisms ([16] and the references therein). Solution oxidation-reduction potentials (ORP) were monitored *in situ* or measured *ex situ*, using an *E*_H_ electrode (Ionode Model IP1306, Pt|Ag/AgCl_(satKCl)_). The probes were calibrated before each experiment using a series of standard ferrous-ferric sulphate solutions (pH 1.8) with the same total iron concentration. Experimental Nernstian constants of RT/nF and *E*° determined at the experimental temperature were used to calculate the ferrous ion concentrations from experimental ORP values using the Nernst Equation (5).

*E* = *E°* − (2.303 RT/*n*F) logQ (5)

where *n* is the number of moles of electrons transferred in the equation, F is the Faraday constant, *E* is the potential difference, *E*° is the standard electrochemical cell potential, R is the ideal gas constant, T is the temperature (degrees Kelvin) and Q is the equation quotient.

**Figure 2:**
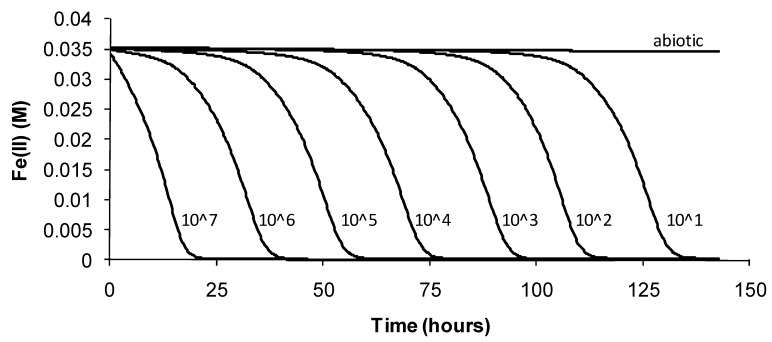
Example data for Fe(II) oxidation to Fe(III) by *At. ferrooxidans* using oxidation-reduction potential (ORP) monitoring as a surrogate measure of cell activity, from which cell growth at 32 °C as a function of time can be inferred. Initial cell densities, prepared by serial dilution, are indicated on the graph.

Corresponding ferric ion concentrations were estimated from the calculated ferrous ion concentrations by the difference from the total iron concentration in the medium. The method is sensitive to cell density (Figure 2) and to total iron concentration [17]. Thus, comparative tests were established using the same initial cell density, and total iron concentrations were measured, especially for tests at pH > 3, where the biologically-generated ferric ion was likely to form insoluble precipitates. Estimated cell doubling times (Dt), lag times (Lt) and final cell densities related to the data in Figure 2 are summarised (Table 1).

**Table 1:** The effects of initial cell density on the growth and activity of *At. ferrooxidans* in basal salt medium (BSM)-Fe(II) (pH 1.8; 32 °C); *n* = 3. Dt, doubling time; Lt, lag time.

Initial Cell Density	Mean Dt (h)	Mean Lt (h)	Final Cells (cells·mL^−1^)
10^7^ cells·mL^−1^	4.4 ± 0.1	3.4 ± 0.2	83 ± 13 × 10^6^
10^6^ cells·mL^−1^	6.0 ± 0.1	18 ± 0.5	70 ± 13 × 10^6^
10^5^ cells·mL^−1^	6.3 ± 0.2	38 ± 3	69 ± 3 × 10^6^
10^4^ cells·mL^−1^	6.4 ± 0.3	55 ± 1	67 ± 12 × 10^6^
10^3^ cells·mL^−1^	5.7 ± 0.8	76 ± 3	69 ± 7 × 10^6^
10^2^ cells·mL^−1^	6.2 ± 0.2	92 ± 4	85 ± 10 × 10^6^
10^1^ cells·mL^−1^	6.4 ± 0.1	114 ± 4	73 ± 3 × 10^6^

### 2.4. Sulphur or Tetrathionate Oxidation

Solution pH was monitored *in situ* in flasks [18] or measured *ex situ* in samples using a calibrated pH meter equipped with a glass membrane electrode. For tests involving microbial growth on elemental sulphur, residual sulphur was estimated by filtering through a 0.2-μm pore size membrane, drying the filtered residue at 40 °C for 16 h and determining mass loss.

**Figure 3:**
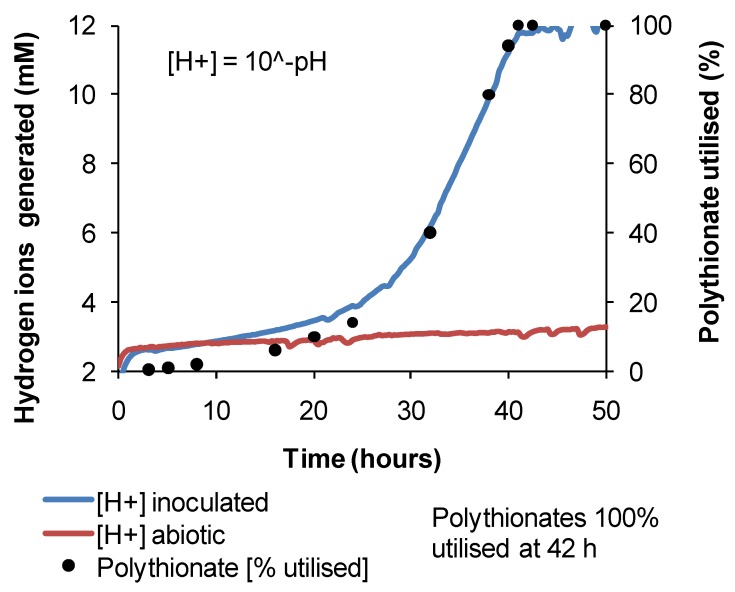
Example data for *At. caldus* grown on tetrathionate at 45 °C, showing the correlation between monitored H^+^ generation (calculated from solution pH) and total polythionate (%) utilisation (data obtained by periodic sampling and HPLC analysis). The slight increase in H^+^ concentration for abiotic medium represents probe drift.

For tests using tetrathionate, pH data were converted to hydrogen ion concentration (Figure 3). During the biooxidation of tetrathionate, other polythionate species, mainly pentathionate, occur as intermediates in the conversion to sulphate [19]. The extent of soluble RISC utilisation was estimated by identifying and quantifying polythionate species in periodic samples using a Waters 2695 HPLC separation module (Waters Limited, Elstree, Hertfordshire, England) using an Ionpac AS16 ion exchange column (Thermo Fisher Scientific, Waltham, MA, USA). Polythionates were detected using a Waters 2996 Photodiode Array Detector (Waters Limited, Elstree, Hertfordshire, England) at 214 nm with the exception of trithionate, detected at 192 nm [20]. A pump flow rate of 1.5 mL·min^−1^ was used, and the column temperature was maintained at 25 °C. A 0.15 M sodium perchlorate solution was used as the eluent. Spectra were integrated using “Empower” software. The relationship between proton generation and sulphate formation during tetrathionate utilisation was linear over the experimental pH range and was used to calculate the % total polythionates utilised (Figure 3). Increased solution ionic strength reduced the kinetics, but not the linearity of this relationship.

### 2.5. Temperature Variation and Substrate Utilisation

The effects of temperature on the rates of Fe(II) or tetrathionate oxidation were determined using a temperature gradient incubator [17] and the data treated using the Ratkowsky equation [21]. The experimental protocol to determine the viability of microorganisms exposed to higher than optimum temperatures entailed transfer of cultures between two shaking incubators controlled at different temperatures. Replicate cultures were prepared in BSM-YE media containing Fe(II) and placed in an incubator controlled at 10 degrees higher than the estimated optimum temperature (T_OPT_) for growth of the test species. Subsequently, flasks were transferred periodically to a second incubator controlled at T_OPT_ where Fe(II) or tetrathionate utilisation was monitored electrochemically using the methods described above.

### 2.6. Dehydration and Recovery

Dehydration tests were conducted by transferring 1.0 mL inoculum into a 2-mL Eppendorf tube (Eppendorf AG, Hamburg, Germany), centrifuging at 14,000 rpm for 10 min, withdrawing the supernatant and then drying the cell pellet in an oven at 40 °C overnight, sealing and storing at 4 °C. Periodically, 1 mL iron(II) or tetrathionate growth medium with yeast extract as needed was added to a tube to resuspend the cell pellet, and duplicate 500-μL aliquots were added to 5 mL fresh medium in 10-mL tubes. The ORP and pH were recorded. Rehydrated cells were incubated at the preferred temperature for the test species. After one week of incubation, solution ORP or pH was measured for Fe(II)- and RISC-oxidation tests, respectively, and cells were counted. Tests were then subcultured and incubated for a further week to confirm growth. Tests were also subcultured into basal salts medium with chalcopyrite concentrate (10% solids loading).

### 2.7. Solution Composition

The ORP- and pH-monitoring methods described above were used to examine the effects of cations and anions on bacterial activity, specifically on Fe(II) or RISC oxidation. In addition, a batch-test method was used for comparative tests on cation inhibition of Fe(II) oxidation. Briefly, these tests were conducted in microtitre plates containing Fe(II) growth media with individual cations at different concentrations [17]. Tests were inoculated to give an initial concentration of 10^7^ cells·mL^−1^. Bacterial growth at each cation concentration was estimated after 7 days by cell counting. Bacterial growth was verified by subculturing from each test into equivalent fresh media and estimating growth after a further seven days. A positive test was recorded if growth occurred in both 7-day cultures. The salts and range of concentrations used were (g·L^−1^): CuSO_4_·5H_2_O (0–250); NiSO_4_·6H_2_O (0–260); CoSO_4_·7H_2_O (0–280); ZnSO_4_·6H_2_O (0–270); and Na_2_HAsO_4_·7H_2_O (0–311). Initial cell numbers were 5 × 10^6^ cells·mL^−1^.

### 2.8. Cell Density

**Figure 4:**
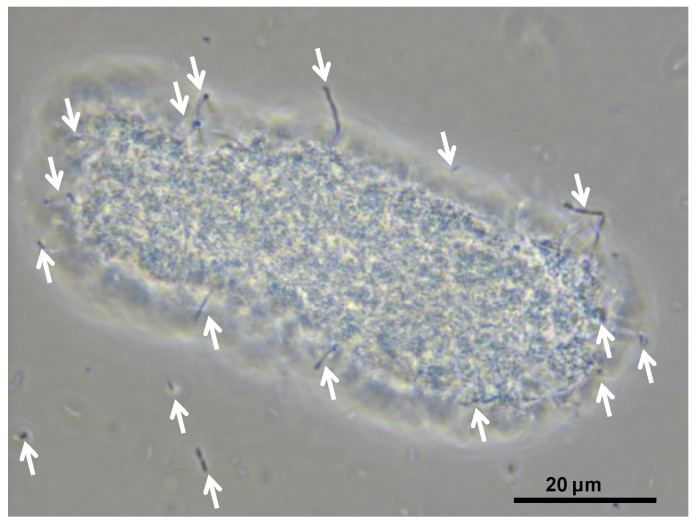
Cells associated with a “particle” of a porous Fe(III) hydroxysulphate precipitate that formed during Fe(II) biooxidation using “*Ab. cupritolerans*”. Cells, some as chains, are indicated by arrows.

Planktonic cells were either enumerated using a Helber Bacterial Counting Chamber (Hawksley, Lancing, Sussex, England) (Thoma ruling, 5.0 × 10^−5^ mm^3^ chamber volume) mounted on a Nikon model “Eclipse Ci” phase-contrast microscope (Nikon Corporation, Tokyo, Japan), or estimated turbidimetrically (OD600) using a Cary 1E UV-VIS spectrophotometer (Agilent Technologies, Santa Clara, CA, USA). Cells in Fe(II)-oxidation tests could be obscured by the formation of Fe(III) hydroxide precipitates, making cell enumeration inaccurate (Figure 4).

## 3. Results and Discussion

### 3.1. Selection of Parameters for Study

**Figure 5:**
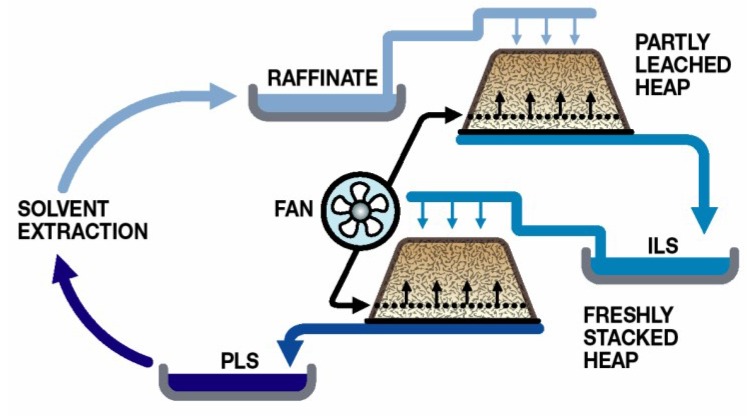
Simplified heap schematic showing solution management and heap aeration to promote the bioleaching of sulphide minerals. ILS, intermediate leach solution; PLS, pregnant leach solution. FAN, low-pressure air blower. Reprinted with permission from [1].

Heap (dump and *in situ*) leaching technologies were developed for the processing of those ores where the metal values are too low or otherwise not suited to flotation concentration and higher-intensity pyrometallurgical processing. Thus, heap leaching is mainly applied to low-grade ores, such as secondary copper sulphides [22], pyritic gold ores [23] or nickel/nickel-copper sulphide ores [24]. The hypothetical heap operation in Figure 5 illustrates the application of solution irrigation, common to both oxide and sulphide ores and of aeration, usually applied to sulphide ores to encourage microbial activity, with solution recycled through a solvent extraction plant where copper is removed from the process water.

Managed leaching heaps are constructed on such a large scale that it is inevitable that ore mineralogy will vary spatially and temporally, no matter the care that is taken to maximise heap uniformity during the construction process.

- Ore mineralogy, mineral associations and liberation determine the degree and rate of heat generation in a heap and, together with climate, influence the duration of above ambient temperatures.
- Ore mineralogy determines acid consumption and solution acidity in different parts of the heap. The application of acid agglomeration ensures more even wetting and acidification of the ore before stacking and reduces the development of dry areas within the bed. The common use of drip-emitters arranged in a relatively wide-spaced grid across the top of the heap does not always result in uniform process water percolation throughout the bed.
- Ore mineralogy, together with the applied acid, determines the process water composition and ionic strength. The recycling of process water in a heap operation results in the build-up of those elements not removed in downstream processes and not incorporated into secondary precipitates.

All of the above variables impact the microorganisms that colonise the ore bed and may or may not remain active for the duration of the leach. While it is extremely difficult to obtain data on microbial behaviour within an operating heap, without disrupting metal production, the data presented in this study help to describe and, in some cases, quantify the responses of microorganisms to heap environments.

### 3.2. Microbial Responses to Variable Temperature

Mineral sulphides are known to oxidise exothermally, mainly due to the contents of pyrite (FeS_2_) or pyrrhotite (Fe_1–x_S, where *x* = 0–0.17). Heat generation is an important parameter in managed sulphide heaps, because some sulphide minerals exhibit strongly increased dissolution kinetics with increased temperature, thus enhancing metal productivity. For example, the secondary copper sulphide chalcocite (Cu_2_S) is oxidised by ferric ions in two stages (Equations (6) and (7)), the first being a rapid reaction that delivers approximately half of the copper to solution. The subsequent much slower oxidation of the “CuS” secondary product (*blaubleibender*; Equation (7)) is strongly temperature dependent; for every ten-degree rise in temperature, the intrinsic rate of “CuS” oxidation increases three-fold [25]. The oxidation of chalcopyrite (CuFeS_2_) is similarly temperature dependent, exhibiting a five-fold increase in copper extraction at 65 °C compared with 35 °C.

Cu_2_S + 2 Fe^3+^ → Cu^2+^ + “CuS” + 2 Fe^2+^ (6)

“CuS” (*blaubleibender*) + 2 Fe^3+^ → Cu^2+^ + S° + 2 Fe^2+^ (7)

**Figure 6:**
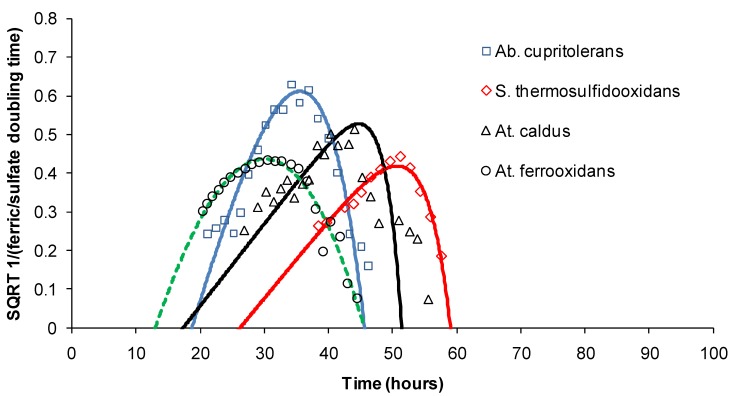
Ratkowsky plots showing the relationships between temperature and ferric-ion generation (“*Ab. cupritolerans*”, *S. thermosulfidooxidans* and *At. ferrooxidans*) or sulphate generation (*At. caldus*) under the experimental conditions used in this study. SQRT = square root.

Heat generation in sulphide heaps impacts the microbial communities that colonise the ores and catalyse the oxidation reactions. It is well known that different bacterial species have defined temperature ranges (“operating windows”) within which they grow well and are active. The ferric-ion generation “doubling times” for “*Ab. cupritolerans*”, *S. thermosulfidooxidans* and *At. ferrooxidans* grown on ferrous ion and the sulphate-ion “doubling times” for *At. caldus* grown on tetrathionate ion obtained for a range of temperatures were modelled using the Ratkowsky Equation (8),

√(1/time) = *b* (*T* – *T*_MIN_) (1 − exp(*c* (*T* – *T*_MAX_)) (8)

where *b* is the regression coefficient of the square root of growth rate constant versus degrees Kelvin for temperatures below the optimal temperature, and *c* is an additional parameter to enable the model to fit the data for temperatures above the optimal temperature [21] The Ratkowsky plots (Figure 6) generate extrapolated values for the minimum (*T*_MIN_), optimum (*T*_OPT_) and maximum (*T*_MAX_) temperatures for the activities of the four species (Table 2)

The data shown for *S. thermosulfidooxidans* are consistent with those reported by Golovacheva and Karavaiko [11], where it was noted that at the temperature limits of approximately 20 °C or 60 °C, the species was practically inactive.

**Table 2:** Extrapolated values of the cardinal temperatures derived from the Ratkowsky Equation [21] for the activity of four bioleaching microorganisms used in this study.

Microorganisms	Substrate	Extrapolated Values			Fitting Parameters	
		*T*_MIN_	*T*_OPT_	*T*_MAX_	“*b*”	“*c*”
“*Ab. cupritolerans*”	Fe^2+^	18.7	36.4	45.6	0.05924	0.0946
*S. thermosulfidooxidans*	Fe^2+^	26.2	50.7	59.0	0.02024	0.2243
*At. caldus*	S_4_O_6_^2−^	17.3	44.7	51.4	0.02127	0.3522
*At. ferrooxidans*	Fe^2+^	13.0	30.0	45.5	0.01633	0.0110

In a heap colonised with these four species, metal extraction from sulphide minerals could be enhanced compared with chemical leaching in the temperature range 15–60 °C. Franzmann *et al.* [16] noted that, in a typical sulphide heap leach operation subject to increasing temperature, a succession of active mesophilic, moderately thermophilic and thermophilic microorganisms would be expected to contribute to the extraction of metals from sulphide ores in an environment.

An important aspect of heat generation in heaps is the speed with which heap temperatures increase. Readett *et al.* [3] operated an aerated, irrigated test heap equipped with temperature probes, constructed using siliceous shale ore containing copper oxide and chalcocite. For this heap, heat generation commenced during the curing period and rose rapidly from about 25–30 °C to 70 °C in the first two weeks when aeration, but not irrigation, was applied. In the absence of aeration (Days 19–25), heap temperatures dropped temporarily. Other reported examples of heat generation in commercially-operated heaps include:

- A full scale heap of ROM ore, averaging 0.2%–0.3% Cu mainly as CuFeS_2_, in which exhaust gases from monitored holes were typically 30 degrees Celsius above ambient temperatures, the maximum temperature (66 °C) occurring between a 6–12-m depth [26].
- An irrigated heap of pyrrhotite-rich copper-nickel sulphide ore, in which temperatures increased to 80 °C within a few days of the commencement of aeration [27,28].
- Non-aerated chalcocite heaps in which internal temperatures up to 46 °C were measured [5].
- Aerated biooxidation heaps of pyritic gold ore in which temperatures ranged from 25 to 80 °C and into which iron-oxidising thermophiles (archaea) were introduced to facilitate biooxidation at temperatures above those suited to bacteria [23].

Initially, ore parameters, such as ore grade, mineral sulphide reactivity and surface exposure, and bed permeability for both solution and air flows are at their maximum. Not surprisingly, therefore, heap temperatures may pass rapidly through the microbial “operating window” and exceed the temperature at which bacteria are active.

**Figure 7:**
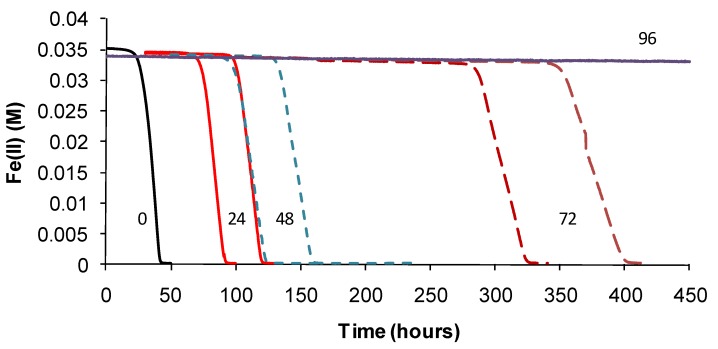
Example data for duplicate heat-stress tests using “*Ab. cupritolerans*” grown in Fe(II) growth medium (pH 1.8). The notations indicate the duration of heat-stress treatments (hours) conducted at 46 °C preceding transfer to a 35 °C incubator.

Based on the above examples, the effect of short-duration exposure to above-optimum temperature on bacterial activity was tested for Fe(II)-oxidising species. Initially, these “heat-stress” tests were conducted at 10 degrees above the *T*_OPT_ of the test species. Subsequently, Fe(II)-oxidising activity at *T*_OPT_ was monitored using the methods described. Periodic examinations of cells were conducted after heat treatment and during monitoring. Example Fe(II)-oxidation data are shown for “*Ab. cupritolerans*” (Figure 7).

The data showed that the primary effect on bacterial activity was to prolong the lag time before Fe(II) oxidation commenced once optimal conditions were restored, from approximately 25 h with no heat treatment to 330 h after 72 h of heat treatment. The recovery time for a 96-h treatment 10 degrees Celsius higher than the optimum temperature for growth was more than three weeks. Estimated mean Fe(III)-generation doubling times increased from 4 h (no heat treatment) to 9 h for the heat-stress tests of a 72-h duration. While cell numbers were not estimated in these tests, periodic examination of cells showed that neither the heat treatment nor the subsequent “optimum” conditions promoted spore formation. Note that Fe(II) oxidation did not commence within three weeks for heat-stress tests of a duration of 96–168 hours, for this species.

**Figure 8:**
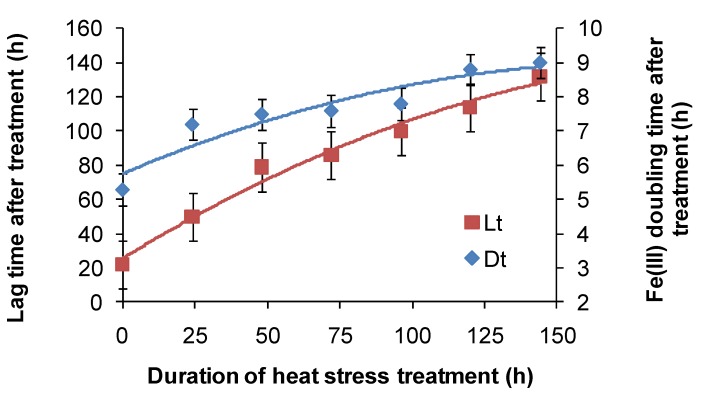
Variability in lag times (Lt) and Fe^3+^ generation doubling times (Dt) induced by short-duration heat stress (pH 1.8, 42 °C) for *At. ferrooxidans* after restoration of optimal conditions (pH 1.8, 32 °C).

For *At. ferrooxidans*, not a spore forming species, Fe(II)-oxidation heat-stress treatments at 10 degrees above *T*_OPT_ for up to 144 h generated similar data. Both lag times and Fe(III) doubling times increased with increased duration of heat-stress treatment, lag times from 20 to 130 h and Fe(III) doubling times from 5 to 9 h (Figure 8). Estimates of final cell numbers for those tests where Fe(II) oxidation occurred were in the range 2 ± 2 × 10^7^–7 ± 1 × 10^7^, but estimate accuracy was compromised by the formation of insoluble Fe(III) hydroxy compounds (see Figure 4). No Fe(II) oxidation occurred after 168 h of treatment within three weeks. Therefore, it is concluded that cell growth was the consequence of Fe(II) oxidation, and the prolonged lag times estimated from these tests were attributed to a large reduction in the number of active cells (see Figure 2), in this case caused by the heat treatment.

**Figure 9:**
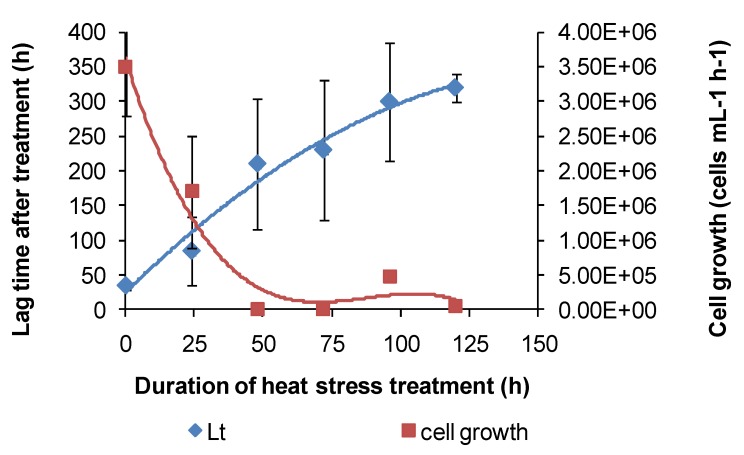
Variability in lag times (Lt) and cell growth rates of *At. caldus* after heat-stress treatments; the reduction in pH was used as an indicator of tetrathionate utilisation.

Similar heat-stress tests were conducted using *At. caldus* grown on tetrathionate, using acid production as a guide to microbial activity. The results indicated that *At. caldus* would be particularly sensitive to increased temperatures in heaps of sulphide ores. Individual lag times were extremely variable, but overall, the trend was towards increased lag times with increased periods of heat stress (Figure 9). Likewise, cellular growth at 45 °C, following short-term heat treatments at 55 °C, was erratic between treatments and among replicates of the same treatment.

Some larger-scale studies in heaps or unsaturated columns (simulating heap conditions) have been undertaken to examine the effect of increased temperature and heat stress on microbial populations. Brierley [29] inoculated a variable-temperature column charged with pyritic gold ore with a mixed consortium of mesophilic and moderately thermophilic bacteria and hyper-thermophilic archaea. Brierley [29] reported that the hyper-thermophiles did not increase in numbers until the column was heated to > 50 °C, but that the moderate thermophiles, such as *S. thermosulfidooxidans*, were detected in all columns up to 60 °C. In isothermal columns charged with low-grade CuFeS_2_ ore and operated at different temperatures up to 60 °C, Mutch *et al.* [30] detected *At. caldus* and a *Sulfobacillus* strain at temperatures up to 50 °C, but surprisingly, the hyper-thermophiles included in the inoculum did not colonise even the 60 °C column. In both of those studies, the presence of mesophilic or moderately thermophilic bacteria in leachates of high-temperature columns was attributed to solution recycling through ambient-temperature reservoirs more amenable to their growth. Halinen *et al.* [31] inoculated isothermal columns containing polymetallic black schist ore with a mixed-microbial consortium enriched from mine water. In the 50 °C column, the bacteria detected in leachate or leach residue included *At. ferrooxidans* and *At. caldus*, both prevalent initially during leaching, when the sulphide content of the ore was at its greatest, and a *Sulfobacillus* species that was detected after 200 days of leaching and prevailed thereafter.

A possible effect of self-heating in heaps is dehydration of microbial cells. Under dehydrating conditions, the ability to form spores should confer a significant advantage. In parallel with the heat-stress tests, *At. ferrooxidans* and *At. caldus* dehydrated and stored for up to six weeks were grown successfully when rehydrated in mixed Fe(II)-tetrathionate growth media or with chalcopyrite concentrate, demonstrating a natural resilience to the short-term effects of dehydration. Similarly, heat-dried cells of *S. thermosulfidooxidans* attached to CuFeS_2_ were revived successfully after four months, with high growth (>5 × 10^7^ cells·mL^−1^) a week after transfer to mixed Fe(II)-tetrathionate growth media. However, isolates obtained from dry samples from a “hot” heap after 2–3 years without irrigation were limited to strains closely related to *S. thermosulfidooxidans* and *At. caldus*, and the bacteria “revived” from heap samples after four years were all strains of *S. thermosulfidooxidans*, indicative of their superior ability to survive for long periods in inhospitable hot and/or dry conditions [17]. The results are consistent with the report that spore formation in *S. thermosulfidooxidans* was strongest when cultures were grown on sulphide minerals and that cultures could survive heat treatments at 100–110 °C [11].

On the basis of these studies, it is concluded that the self-heating of sulphide heaps poses a risk to successful microbial colonisation. Short-term heat-stress treatments at temperatures 10 degrees Celsius above the optimum temperatures for the growth of particular microbial strains caused prolonged periods of inactivity, and recovery from week-long heat treatments was extremely poor. The large masses of ores in bioleaching heaps mean that high temperatures arising from sulphide oxidation are hard to control initially, while the sulphide content of the ore is at its greatest. During that period, the numbers of mesophilic and moderately thermophilic bacteria will be markedly reduced in both numbers and activity. Recovery of a microbial population from prolonged heat stress will require re-inoculation via cells in the cooler process water being fed to the heap surface and, with prolonged detrimental hot, dry conditions, revival from microbial spores within the ore bed.

### 3.3. Microbial Responses to Variable Process Water Acidity (pH)

**Figure 10:**
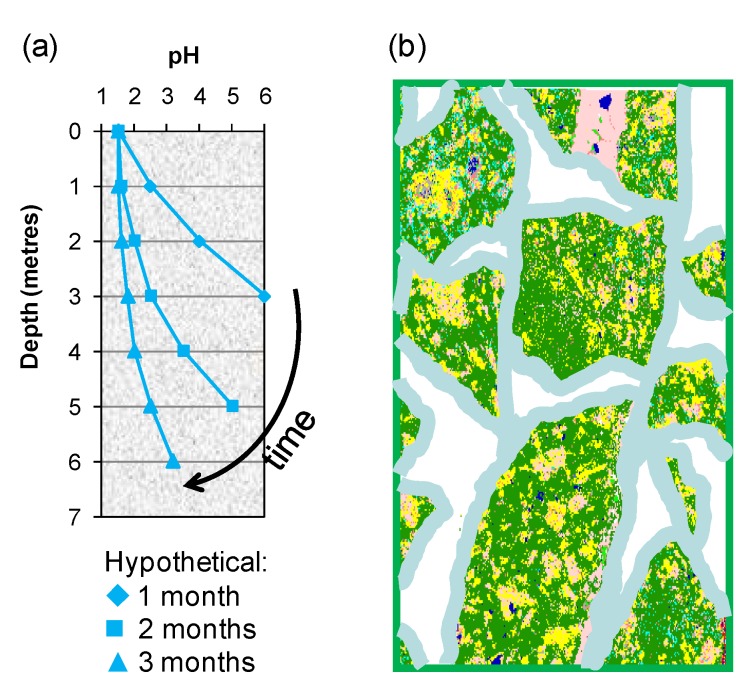
Schematic illustrating (**a**) the acid front moving through a porous ore bed and (**b**) polymineralic particles covered with a thin film of moisture within which chemical and microbial reactions take place.

Two of the means by which variable process water acidity may arise in heaps are: (i) gangue-mineral acid consumption as the solution percolates through the bed from top to bottom, a bulk effect in which an “acid front” eventually reaches the base of the heap (Figure 10a); and (ii) individual solution contact with particle surfaces that creates micro-environments differing in acidity from the bulk solution in the unsaturated bed (Figure 10b).

It is thought that the population of acidophilic sulphur and Fe(II)-oxidisers will migrate through the ore bed with the acid front. Bacterial oxidation of Fe(II) requires a pH < 2, because in more alkaline solutions, the rate of chemical oxidation of Fe(II) to Fe(III) increases significantly and competes with biological Fe(II) oxidation. The Fe(III) generated forms insoluble compounds, thus removing the growth substrate from the solution. Halinen *et al.* [32] operated unsaturated pH-controlled columns of polymetallic black schist ore in the range pH 1.5–3.0 and concluded that pH 2 was optimum for good metal extraction and would minimise excess acid consumption due to gangue mineral dissolution. Tupikina *et al.* [33] reported that irrigation of unsaturated columns with a solution pH 1.7 or higher resulted in loss of the soluble iron, but with a solution feed of pH 1.4, there was a net increase in soluble iron.

It is not surprising, therefore, that Fe(II) oxidation by different bacteria occurs over a limited pH range (approximately 0.5–2.5) [32], outside which those bacteria only able to oxidise Fe(II) will become inactive. The dual ability of *At. ferrooxidans* to grow on Fe(II) and/or RISC is well established. The reported pH range for growth is pH 0.8–6 with an optimum of pH 1.8–2 and probably represents the range for RISC oxidation [33]; the reported range for Fe(II) oxidation of pH 1.3–4.5 (optimum pH 2.5) [34] would be expected to be somewhat narrower, but may have included a contribution from chemical Fe(II) oxidation at the high end of the range.

**Figure 11:**
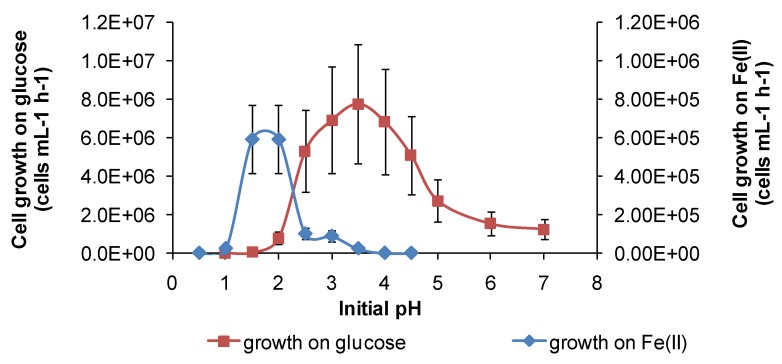
Growth rates of “*Ab. cupritolerans*” in media of different initial pH, with Fe(II) (30 °C, 168 h, *n* = 5) or glucose (35 °C, 72 h, *n* = 3) as substrate. Initially, 10^6^ cells·mL^−1^.

**Figure 12:**
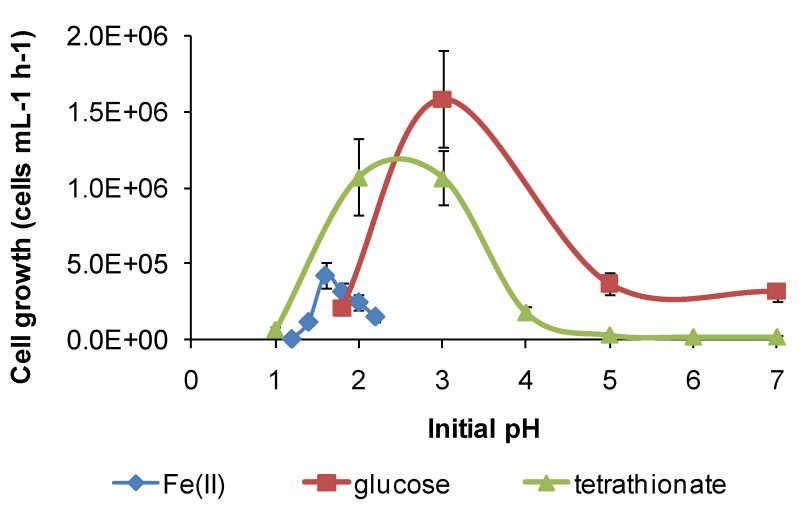
Growth rates (cells·mL^−1^·h^−1^) of *S. thermosulfidooxidans* in media containing iron(II), glucose or tetrathionate as substrate at 50 °C with different initial pH. Initial cell concentration, 10^6^ cells·mL^−1^. Duration of test, 168 h.

Under the conditions used in the present study, “*Ab. cupritolerans*” oxidised Fe(II) in the range of pH 1–2.5 (Figure 11) and *S. thermosulfidooxidans* oxidised Fe(II) in the range of 1.2–2.2 (Figure 12). Microbial Fe(II)-oxidation rates declined rapidly outside those limits. The data obtained for Fe(II) oxidation for *S. thermosulfidooxidans* were consistent with those reported by Plumb *et al.* [34], with maximum growth at pH 1.5.

However, for both species, there was a broader pH window for growth when organic substrates were metabolised, for example glucose (Figure 11 and Figure 12), ensuring bacterial survival at solution pH where soluble Fe(II) is not available. The ability of *S. thermosulfidooxidans* to grow on organic compounds after a short period of adjustment was reported, as was the enhanced leaching of sulphide ores in the presence of added glucose [11], but the broadening of the pH window for growth (Figure 12) was not previously noted. In addition, for *S. thermosulfidooxidans,* RISC oxidation occurs in solutions of pH 1.2–3.6, providing a third metabolic strategy for survival in variable heap environments. The growth of *S. thermosulfidooxidans* on sulphur in controlled pH experiments was reported to occur in the range of pH 2–4.5 [11].

**Figure 13:**
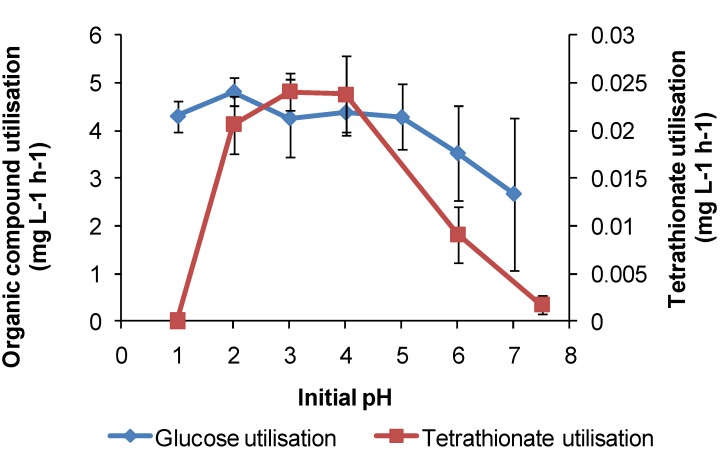
Utilisation rates (mg·L^−1^·h^−1^) for *At. caldus* growth on tetrathionate or glucose as substrate at 45 °C in media with different initial pH. Initial cell concentration, 2 × 10^7^ cells·mL^−1^; maximum duration of tests, 168 h (*n* = 3).

For *At. caldus* in tetrathionate medium, cell growth occurred in the range of pH 1.5–5, with a maximum cell generation rate of 4 × 10^6^ cells·mL^−1^·h^−1^ at pH 3. Tetrathionate utilisation occurred in the range of pH 1.5–6 with a maximum generation rate in the range of pH 3–4 (Figure 13). The maximum growth and tetrathionate utilisation measured in this study at pH 3 was slightly higher than the optimum pH 2–2.5 reported previously [12] or the optimum pH 2 for growth on elemental sulphur [34]. *At. caldus* also grew on glucose (with 0.1 mM tetrathionate) across pH 1–7 at 45 °C, with the optimum at approximately pH 2, again demonstrating a possible alternative metabolism if RISC were absent in a heap. The requirement for RISC to be present for growth on glucose by *At. caldus* was reported by Hallberg and Lindström [12].

The results of some larger-scale studies on microbial stress induced by acidity have been published, but these tests, inoculated with mixed microbial cultures, were directed towards increased acidity in the region of pH 1 or lower (e.g., [32,35]), rather than under conditions of gangue mineral acid consumption and solution pH rising above pH 2. The results shown in Figure 11, Figure 12 and Figure 13 are consistent with microbial activity being much reduced when the solution conditions approach pH 1 or less, as reported in the cited studies.

In summary, each of the study species has at least two metabolic pathways with different solution pH ranges for growth that could be activated in solutions of variable acidity. It appears that the restricted range of solution acidity for Fe(II) oxidation is largely determined by the solubility of Fe(III) and Fe(II) species. The two examples from this study (Figure 11 and Figure 12) both indicate optima for Fe(II) oxidation at pH < 2. At approximately pH 2 and higher, chemical oxidation of Fe(II) occurs in the presence of oxygen, and Fe(III) hydroxy compounds are precipitated and rates increase rapidly with increased pH. RISC oxidation occurs over a broader range of solution acidity, as does organic carbon utilisation (Figure 11, Figure 12 and Figure 13). At pH < 1, microbial activity, whichever metabolism is employed, is curtailed by the need to restrict protons from entering the cells, facilitate acid tolerance and/or reduce the effects of acid on cells. A number of mechanisms employed by microorganisms to overcome the effects of acid have been reviewed [36], but are outside the scope of this work.

### 3.4. Microbial Responses to Process Water Components

The majority of heap leaching operations use sulphuric acid as the lixiviant and rely upon microbial Fe(II) oxidation to generate the ferric ions necessary for sulphide oxidation. Process water management is directed towards minimal water use and involves recycling to the heaps after the removal of the target metal (e.g., Cu; Figure 5). While some recent studies have examined the effects of low-pH process water on microbial colonisation of ores (e.g., [35]), it is a reality that the application of low-pH water to heaps results in greater acid consumption, a major cost, and in increased gangue mineral dissolution, resulting in high total dissolved solids process water [30]. Element concentrations in heap process waters can build to high concentrations, although, in the case of the data compiled from different case studies (Table 3), only one or two of the elements were simultaneously at the upper concentration, depending on the ore being leached. For heaps irrigated with seawater, chloride in process waters strongly inhibits microbial activity, as do fluoride and/or nitrate leached from some ores. Acidophiles that colonise managed heaps develop resistance to heavy metals that provide them with a competitive selective advantage [37].

**Table 3:** Approximate upper limits (g·L^−1^) for individual cations/anions in heap process water.

Cu	Ni	Co	Zn	As *	Fe	Mg	Al	SO_4_	Cl ^#^	F	NO_3_
6	5	<1	23	20	25	10	25	130	20	2	35

* Upper limit maintained by operator to avoid microbial inhibition; ^#^ assuming no evaporation and no additional chloride contribution from ore minerals.

Methods used to investigate metal tolerance of microorganisms vary. Some rely only on the increase in cell numbers for a given time period, and others measure microbial activity during growth on a substrate. Initially, metal tolerance data for the four study species were obtained using the batch method (Section 2.7) and estimating cell numbers after 168 h. The results (Table 4) indicated that there was great variability between replicate estimates using the same strain and also between strains from different heaps. A cursory review of the literature showed that metal toxicity data for *At. ferrooxidans* (e.g., [38,39,40]) similarly exhibits a high degree of variability within and between studies.

**Figure 14:**
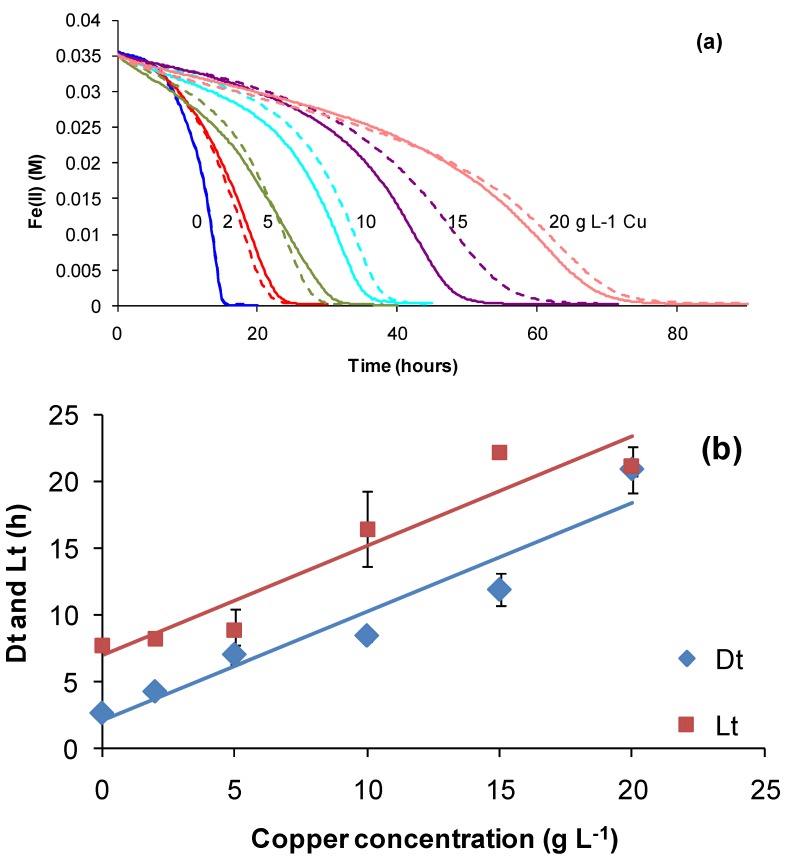
Fe(II) oxidation by *At. ferrooxidans* in the presence of copper: (**a**) effect of copper (duplicate tests); the initial Cu concentrations are noted on the graph; (**b**) estimated Fe(III)-doubling times (Dt) and lag times (Lt) (*n* = 2).

Subsequently, in experiments to explore the variability further, metal tolerances were examined using the ORP- and pH-monitoring methods described for Fe(II) and RISC oxidation, supplemented by estimates of cell growth. Example data are given for the effect of copper on Fe(II) oxidation by *At. ferrooxidans*. The main effect of copper was to slow Fe(II)-oxidation rates progressively with increased concentration (Figure 14a), rather than to prolong the lag time markedly (Figure 14b). The data are interpreted as indicating that cells did not become completely inactive, but performed their functions more slowly in the presence of Cu.

**Table 4:** Ranges of metal tolerances estimated from batch tests after 168 h for “*Ab. cupritolerans*”, *At. ferrooxidans*, *At. caldus* and *S. thermosulfidooxidans* and some closely-related isolates obtained from copper heaps.

Species	Element (g·L^−1^)				
	Cu	Ni	Co	Zn	As
“*Ab. cupritolerans*”, from heap, adapted	175	15	15	60	15
*At. ferrooxidans*, unadapted	65	225	140	285	45
and related isolates from heap, adapted	75	265	170	285	60
*At. caldus*, unadapted	1–5	2–15	5–25	65–285	30
and related isolates from heap, adapted	5–25	5–50	5–50	50–185	60
*S. thermosulfidooxidans*, unadapted	20–75	9–40	1–2	15	15
and related isolates from heap, adapted	15–50	5–165	1–10	10–50	15

**Figure 15:**
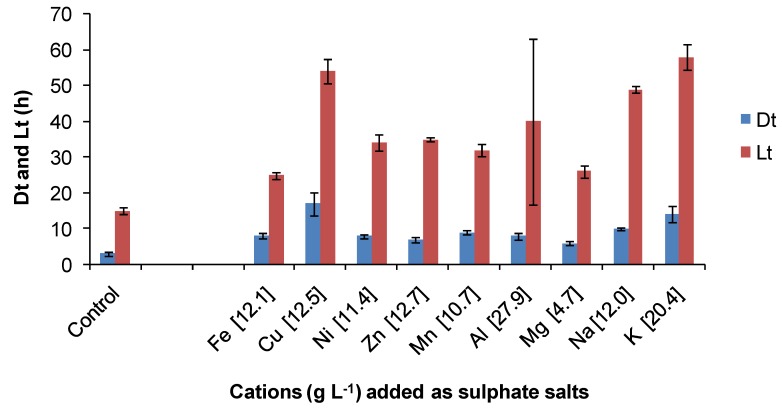
Comparative effects of some cations on Fe(II) oxidation by *At. ferrooxidans* in media of 1 M ionic strength. Cations (g·L^−1^) are indicated on the X axis. Dt, ferric ion generation doubling time; Lt, lag time; initial cell concentration, 10^6^ cells·mL^−1^; maximum duration of tests, 168 h, or less if the substrate was fully utilised (*n* = 3).

**Figure 16:**
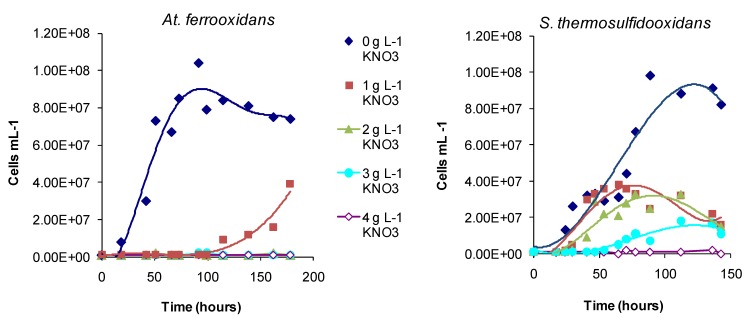
Effect of nitrate (0–4 g·L^−1^) in mixed Fe(II)-tetrathionate medium, on the growth of *At. ferrooxidans* and *S. thermosulfidooxidans*. Tests conducted at 30 and 50 °C, respectively.

The same ORP-monitoring method was used to compare the effects of several contaminant cations and the anions chloride and sulphate (Figure 15). In these tests, the control contained 35 mM of Fe(II) in BSM (Fe-BSM ionic strength 0.22 M) and amounts of contaminant elements were added to yield media with final ionic strength 1 M. While it may be considered that an ionic strength of 1 M is “high”, values from 1.4 to 7.6 M have been estimated from heap process water compositions ([7] and the references therein), largely influenced by Fe(III), Al(III) and SO_4_ concentrations. In the presence of cobalt sulphate (Co 10.7 g·L^−1^), the Fe(II) oxidation rate was very slow, preventing the estimation of Dt; the lag time was estimated to be 108 hours. There was no Fe(II) oxidation in the presence of sodium chloride (Na 17.9 g·L^−1^) in the time frame of the experiment (168 h). The particular sensitivity of acidophiles to chloride involves complex, pH-dependent mechanisms that result in the acidification of cell cytoplasm when the growth environment has low pH [41]. This sensitivity restricts the use of seawater (0.5 M chloride) or more concentrated saline bore water for leaching operations [7]. A comparative study using a batch-culture technique with Fe(II) or tetrathionate as the substrate showed that *At. ferrooxidans* grew in medium with up to 7 g·L^−1^ NaCl, *At. caldus* with up to 15 g·L^−1^ NaCl and *S. thermosulfidooxidans* with up to 7 g·L^−1^ NaCl [42]. Thus, the expectation was low that growth at up to 1 M NaCl (58 g·L^−1^) would occur in this comparative study (Figure 15). When sodium nitrate was added as the contaminant, the Fe(II) was oxidised chemically before inoculation, nullifying the test. In the tests illustrated in Figure 16, estimated Fe(III) doubling times and lag times were greater than those estimated for the control (lower ionic strength). On the basis of the estimated lag times, it was concluded that the most inhibitory cations tested were cobalt > copper and that chloride was more inhibitory than sulphate. With the exceptions of cobalt and chloride, Fe(II) oxidation by *At. ferrooxidans* occurred in 1 M ionic strength synthetic “process water” containing a single contaminant.

**Figure 17:**
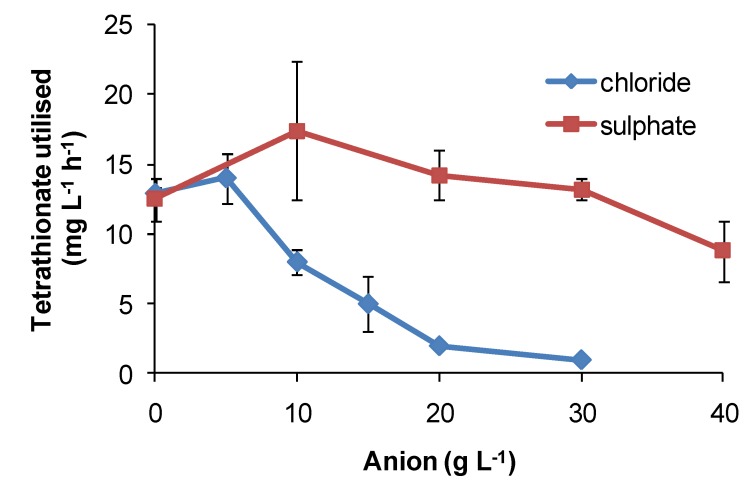
Effect of sulphate and chloride anions added as sodium salts on rates of tetrathionate utilisation (mg·L^−1^·h^−1^) by *At. caldus* at 45 °C. Initial cell concentration, 10^6^ cells·mL^−1^; rate data estimated over 100 h, or less if substrate fully utilised (*n* = 2).

Regarding the effects of anions, in comparative one-week tests using *At. ferrooxidans* or *S. thermosulfidooxidans*, the presence of nitrate caused slower Fe(II)- and tetrathionate-oxidation with increased lag times and reduced cell numbers (Figure 16) relative to nitrate-free tests. Chloride was more inhibitory than sulphate for *At. caldus* grown in tetrathionate medium (Figure 17). The rank order was nitrate > chloride > sulphate.

**Figure 18:**
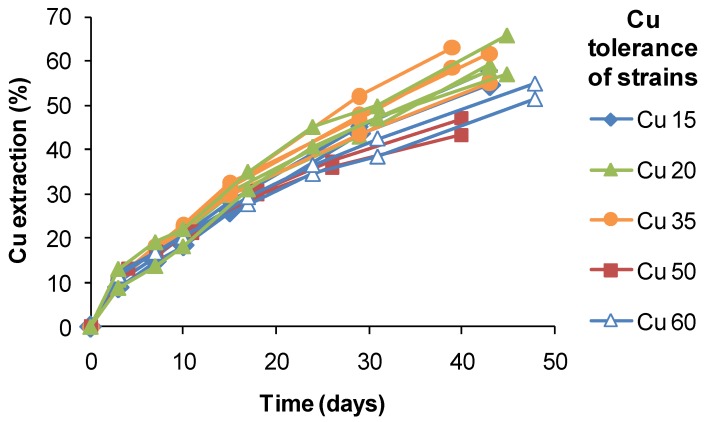
Copper extraction by native strains of *S. thermosulfidooxidans* from copper sulphide heaps. Different strains had tolerances of up to 60 g Cu L^−1^. Test conditions were: initial pH 1.8, 1% w/v CuFeS_2_ concentrate, 45 °C, leach duration 30–48 days; test leach solutions did not exceed 5 g Cu L^−1^.

In general, the data presented in Table 4 provided evidence of adaptation to the metals by some isolates obtained from the heaps, compared with the Deutsche Sammlung von Mikroorganismen und Zellkulturen (unadapted) strains of the same species. The data also showed that the four strains exhibit tolerances to Cu, Ni, Co, Zn and As that would allow them to oxidise Fe(II) in heap process waters close to or above the upper cation concentrations in Table 3. While the benefits of adaptation have not been investigated in this study, it is assumed that adapted strains would exhibit reduced lag times and/or faster Fe(II) or RISC oxidation rates compared with “controls”. This assumption is based on the results of prolonged exposure to 4-nonylphenol, a minor, but strongly-inhibitory component of a solvent extraction reagent commonly used in the separation and purification of copper from heap process water. Using the electrochemical Fe(II)-oxidation monitoring method, Collinson *et al.* [43] demonstrated for *S. thermosulfidooxidans* that “near normal” Fe(II)-oxidation rates and lag times were restored after a year of adaptation to low concentrations of 4-nonylphenol. Similarly, while *At. ferrooxidans* and *S. thermosulfidooxidans* were shown to be inhibited by nitrate in Fe(II) growth medium, in bioleaching tests (30 °C or 45 °C) with CuFeS_2_, the mixed culture of bacteria and archaea, including *At. ferrooxidans* and *S. thermosulfidooxidans*, adapted to the presence of nitrate within nine weeks [44]. In a related study using *S. thermosulfidooxidans*-like isolates from a copper heap, the range of extractions obtained after 40 days of leaching varied by approximately 20%, but did not correlate with estimated Cu tolerances (Figure 18). It is possible that the slightly lower extractions obtained using strains tolerant to 50–60 g Cu L^−1^ were a result of slower Fe(II) oxidation and limited Fe(III) production. Therefore, while attributing the relatively high Cu tolerances to adaptation to the ore, adaptation itself did not directly confer an enhanced ability to extract Cu from CuFeS_2_ in bioleaching tests.

Nevertheless, adaptation has played a role in commercial bioleaching. In a recent review [45], examples of microbial adaptation were identified in a number of pilot-, demonstration- and commercial-scale, stirred-tank plants and from the substantial test programmes. Such descriptions were scarce, but included microorganisms that adapted to 17–20 g·L^−1^ Cu, 6–7 g·L^−1^ Zn, 13–15 g·L^−1^ Fe and 65–70 g·L^−1^ sulphate when grown on a polymetallic concentrate [46]. In a stirred-tank pilot plant for the oxidation of nickel sulphide concentrate, the microorganisms grew in the presence of 23 g·L^−1^ Ni and 38 g·L^−1^ Fe [47]. During the development of a commercial-scale, stirred-tank process for the extraction of cobalt from pyritic tailings, microorganisms grew in the presence of >5 g·L^−1^ Co and > 35 g·L^−1^ Fe [48], and mineral oxidation was at least 30% faster in continuous reactors than in batch reactors [49]. Continuous culture of *At. ferrooxidans* on arsenopyrite for one year resulted in cultures able to grow in the presence of 5 g·L^−1^ arsenite and 30 g·L^−1^ arsenate [50], and the presence of arsenic-resistance genes in *S. thermosulfidooxidans* [51] could explain its prevalence (with other *Sulfobacillus* spp.) in several continuous bioreactors processing refractory gold concentrates [52].

## 4. Conclusions

Customised monitoring methods for the three main bacterial functions, ferrous-ion oxidation, sulphur oxidation and carbon utilisation, have been developed and example test data presented for up to four bacterial species found in copper sulphide heaps. The variables selected represent three key characteristics of heap environments: the consumption of acid leading to localised areas of low solution acidity, the impacts of solution components derived from the dissolution of copper and/or gangue minerals in recycled process water and fluctuating temperatures due to sulphide oxidation.

The results indicate that each of the study species has at least two metabolic pathways with different solution-pH ranges for growth that can be engaged in environments with variable acidity. The restricted pH ranges described for many bacteria capable of Fe(II) oxidation are largely determined by the solubility of Fe(III) and Fe(II) species. RISC oxidation occurs over a broader range of solution acidity, as does organic carbon utilisation. At pH < 1, microbial activity is greatly reduced whichever metabolism is employed. The four strains exhibit tolerances to Cu, Ni, Co, Zn and As that would allow them to oxidise Fe(II) in heap process waters. The data provide evidence of adaptation to the metals by isolates obtained from the heaps. The finding that bacterial metal tolerance does not result in greater enhancement of copper extraction is industrially relevant. The self-heating of sulphide heaps poses the greatest risk to successful microbial colonisation and the consequent enhanced metal extraction. The large masses of ores in bioleaching heaps mean that high temperatures arising from sulphide oxidation are hard to control initially, while the sulphide content of the ore is at its greatest. During that period, the numbers of mesophilic and moderately thermophilic bacteria would be markedly reduced in both numbers and activity.

The results presented form part of an ongoing study to create a database of microbial behaviours in heaps. It is proposed that laboratory-generated data under different conditions, when linked with metallurgical data acquired during heap operation, offer an indirect means of inferring “biological health”. Thus far, strains examined individually have exhibited a variety of responses to the selected solution chemistry parameters. However, in heaps of sulphide ores, the microbial communities will comprise several active microbial strains that may or may not contribute to enhanced metal extraction. A more complete database will require similar studies using mixed cultures subjected to varied, but controlled conditions, as might evolve during heap leaching. The studies would preferably be conducted in continuous reactors and make use of modern DNA-based microbial identification and quantification methods suited to environmental samples to understand microbial population dynamics under adverse conditions.
